# Serum TNF-Alpha and IL-10 Predict Reduced Sensitivity to Fear- and Anxiety-Related Traits in Healthy Older Dogs: Preliminary Evidence for Immune–Personality Signatures in Later Life

**DOI:** 10.3390/ani15162418

**Published:** 2025-08-18

**Authors:** Federica Pirrone, Virginia Bettoni, Mariangela Albertini, Alessia Giordano, Stefania Melzi, Amna K. T. Naji, Simona Nonnis, Patrizia Piotti, Letizia L. M. Schifino, Saverio Paltrinieri

**Affiliations:** 1 Department of Veterinary Medicine and Animal Sciences, University of Milan, via dell’Università 6, 26900 Lodi, LO, Italy; virginia.bettoni@unimi.it (V.B.); alessia.giordano@unimi.it (A.G.); amna.naji@studenti.unimi.it (A.K.T.N.); simona.nonnis@unimi.it (S.N.); patrizia.piotti83@gmail.com (P.P.); letizia.schifino@studeniti.unimi.it (L.L.M.S.); saverio.paltrinieri@unimi.it (S.P.); 2 Ambulatorio Veterinario San Francesco, via Leonardo da Vinci 40, 20021 Bollate, MI, Italy; sanfrancescobollate@gmail.com

**Keywords:** aging, canine personality, canine inflammation, cytokines

## Abstract

As dogs live longer, they face an increased risk of functional age-related changes, including physiological and behavioral alterations. However, data on these age-related processes in domestic dogs remain limited. In this study, we assessed serum concentrations of cytokines (TNF-α, IL-6, IL-10), personality and cognition using validated scales (Dog Reinforcement Sensitivity Theory Personality Questionnaire (RSTQ-D), Dog Impulsivity Assessment Scale (DIAS), and Canine Cognitive Assessment Scale (CCAS)) in 44 healthy pet dogs from two non-overlapping age groups (1–4 years old and ≥11 years old). Generalized linear models revealed small but significant (*p* = 0.001) negative effects of age-by-cytokine interactions in predicting sensitivity to RST personality traits related to fear and anxiety in older dogs. Specifically, higher TNF-α and IL-10 levels predicted lower scores on the Fight/Flight/Freeze System (FFFS) and the Behavioral Inhibition System (BIS). These findings provide novel insights into previously unrecognized associations between the immune system and personality in healthy older dogs, possibly contributing to broader profiles of vulnerability or, conversely, resilience. Further research is needed to confirm these associations and clarify their functional meaning. Such knowledge could facilitate the early identification of complex aging-related phenotypes and inform individualized preventive strategies to support the health and well-being of aging companion dogs.

## Simple Summary

Aging is accompanied by chronic low-grade inflammation, immunosenescence, and age-related diseases. Given its multidimensional nature, there is an urgent need for multimodal and multiperspective approaches in aging research. While evidence from humans and rodents suggests age-related changes in emotional behavior and cognition, findings remain inconsistent, and little is known about these processes in companion dogs. This study explored whether immune markers predict differences in personality traits and cognitive function in clinically healthy dogs from two different age groups and whether these associations are moderated by age, aiming to identify multidimensional links that shed light on the pathophysiology of the aging process. Our results suggest the existence of immune–personality signatures in healthy older dogs, specifically involving serum TNF-α and IL-10 levels in association with fear- and anxiety-related traits. These patterns are not mutually exclusive, and their expression may reflect different functional dynamics, likely depending on individual factors such as life-course exposures. While preliminary, these findings may inform research on healthy aging through longitudinal and mechanistic studies, laying the groundwork for clinical monitoring in senior dogs.

## 1. Introduction

The longevity of pet dogs has increased notably in recent decades, with an average lifespan currently estimated at 12.7 years [1]. This increase, largely attributed to changes in living environments, along with improvements in nutrition, veterinary healthcare, and preventive care [2,3], and potentially influenced by genetic factors related to body size, such as variation in the IGF1 gene [4], has led to a consequent growth in the population of older dogs [5]. As in humans, the extended lifespan is accompanied by an increased prevalence of comorbidities, particularly in older individuals, who experience a state of vulnerability and decline in physiological resilience as a result of frailty [6,7]. The frailty syndrome has been extensively studied in humans, and several contributing factors, such as aging itself, chronic conditions, cognitive decline, and personality traits, particularly higher neuroticism (characterized by nervousness, anxiety, and insecurity), have been identified [8]. Conversely, this condition remain poorly understood in dogs [6]. Given the mentioned implications, a better understanding of the fundamental mechanisms of aging, including the identification of factors associated with frailty, is crucial to improve the well-being of older dogs and to extend not only their lifespan but also their health-span, that is, their life expectancy in good health [6].

Aging profoundly impacts the immune system, involving changes in hematopoiesis, as well as in adaptive and innate functions, which are closely linked to a pro-inflammatory state [9]. This interplay between aging and inflammation is commonly known as “inflammaging” [10] and describes a chronic, low-grade inflammatory state that (1) is closely associated with immune dysregulation (immunosenescence), (2) develops with age, and (3) occurs even in the absence of overt infection. Despite this state being first conceptualized in humans over 30 years ago and later recognized in dogs as well [11,12], the physiology and pathophysiology of inflammaging are complex and not yet fully understood. Basically, immune cells communicate with each other through the production of pro-inflammatory (e.g., TNF-α, IL-1β and IL-6) and anti-inflammatory (e.g., IL-10) cytokines. Senescent immune cells, particularly T cells and macrophages, shift their secretory profile toward a pro-inflammatory phenotype, increasing the release of pro-inflammatory cytokines, while reducing the production of anti-inflammatory ones [13]. Similar findings to those observed in humans, such as age-related increases in IL-6 and TNF-α, have been recently described in dogs. Jiménez (2023) [14] found lower IL-6 concentrations in dogs under 3 years of age compared to older dogs but no differences among older age groups (i.e., 3–6 years, 7–10 years, and 11 + years). However, the lack of medical history or clinical data in the study may have allowed the inclusion of dogs with undiagnosed subclinical conditions, potentially confounding results. A more recent pilot, cross-sectional study by Schmid et al. (2024) [15] used rigorous health screening and found positive associations between age and serum levels of IL-6, IL-8, and TNF-α, along with lower lymphocyte counts. However, as noted by Alexander et al. (2018) [16], evidence of changes in specific inflammaging-related markers in dogs remains preliminary and has primarily focused on pro-inflammatory cytokines. The role of markers involved in resilience capacity, such as anti-inflammatory cytokines, also requires characterization, as does the relationship between both pro- and anti-inflammatory markers and other age-related functional alterations in dogs.

Because the central nervous system is particularly sensitive to inflammaging due to its limited regenerative capacity, with neurodegeneration and cognitive decline representing potential direct consequences [17], functional changes in cognition and behavior are among the most commonly observed alterations in aging dogs [18]. Cognitive domains such as learning, memory, and attention may progressively decline [19,20], ranging from normal aging, which does not impair daily functioning or the pet–caregiver relationship, to mild cognitive impairment and, in some cases, to Canine Cognitive Dysfunction Syndrome (CCDS), a condition closely related to Alzheimer’s disease in humans [21,22], which may go underdiagnosed in the older canine population [23]. Alongside cognitive changes, dogs may also show alterations in personality traits as they age [19,20,24,25], reflecting the complexity of the aging process and its tendency for considerable inter-individual variations. Animal personality refers to individual differences in behavior that are consistent over time and across contexts [26]. Various frameworks have been used to investigate these traits in dogs, ranging from trait clusters such as boldness, sociability, and fearfulness to broader models like the Five-Factor Model (FFM) [27], which has been adapted from human psychology to describe canine traits such as extraversion, neuroticism, and agreeableness. Tools like the Dog Mentality Assessment (DMA), the Behavior and Personality Assessment in Dogs (BPH), and the Dog Personality Questionnaire (DPQ) have all contributed to characterizing individual behavioral profiles [28]. Importantly, personality traits such as boldness and fearfulness have been linked to emotional predispositions and cognitive–emotional processes in dogs, as shown in studies using cognitive bias tests [29]. Specific dimensions of impulsivity, such as response inhibition, and traits like novelty seeking tend to decrease with age [30]. In contrast, aging has been associated with increased expression of both fear and anxiety-related behaviors in dogs [30], patterns also observed in aged rodents and humans and commonly linked to both neurodegenerative and non-pathological aging processes [30,31,32]. It may be worth noting that fear and anxiety are two related but distinct emotional states. Fear is typically defined as a behavioral response to an actual and immediate threat [33], whereas anxiety arises in potentially threatening situations or when certain environmental cues predict a negative outcome [34]. Interestingly, Careau et al. (2010) [35] found a relationship between energy expenditure and proactive personality traits (such as boldness and aggressiveness) in dogs, as well as between longevity and trainability, with shy, long-lived individuals contrasting with bold, short-lived ones, according to a pace-of-life perspective.

These patterns can vary considerably among individuals and may be influenced by subtle or unrecognized age-related impairments, including subclinical or undiagnosed cognitive dysfunction [36]. Given that aging is a gradual process, mild and subtle changes likely indicate an early stage, when targeted interventions may be most effective in slowing progression into true pathological states and in supporting better stress management and adaptation, ultimately helping preserve well-being and quality of life in aging dogs [37].

To the best of our knowledge, no studies to date have examined whether and how personality traits interact with other biological age-related changes, for instance, exploring age-specific, multidimensional associations with immune features in animals who are still free from clinically relevant diseases. The valuable insights gained from exploring these cross-domain relationships across different age groups could help reveal both age-related vulnerabilities and adaptive mechanisms, ideally informing the development of targeted strategies to preserve behavioral health and resilience in aging dogs. Therefore, the purpose of this cross-sectional study was to investigate whether an interplay exists among serum immune markers (TNF-α, IL-6, and IL-10), personality traits, and cognitive function in healthy dogs at two distinct life stages (1–4 years and ≥11 years). Middle-aged dogs (5–10 years old) were intentionally excluded to maximize the contrast between early and late life stages. We hypothesized that cytokines expression would predict variations in behavioral traits and cognitive performance, as a function of age, reflecting immune–behavior dynamics that may emerge during the aging process, even in the absence of clinically relevant diseases. General behavior and personality were assessed using two validated observational questionnaires increasingly adopted in canine behavioral science: the Dog Impulsivity Assessment Scale (DIAS) and the Reinforcement Sensitivity Theory Personality Questionnaire-Dog (RSTPQ-D) [38,39]. These tools were chosen for their relevance in evaluating individual behavioral profiles and their suitability for examining associations with cognitive, emotional, and physiological parameters in both research and clinical contexts [39,40]. Although the DIAS and RSTPQ-D share some conceptual overlap, each captures distinct dimensions of personality [39], justifying their combined use to provide a more comprehensive assessment of dogs’ behavioral profiles. Cognitive function was evaluated using the Canine Cognitive Assessment Scale (CCAS), as modified by the Companion Animal Welfare Education Center [41], which provides a graded classification of cognitive impairment and covers a broad range of behavioral changes [42].

## 2. Materials and Methods

This study was part of a large research project designed to investigate the effects of age-related chronic inflammation on the health and behavior of elderly dogs. Recruitment occurred through opportunistic sampling among the patients admitted to the Veterinary Teaching Hospital of Lodi, University of Milan (OVU UNIMI) for regular vaccinations or minor health issues and from dogs referred for suspected age-dependent behavioral changes. All caregivers signed written informed consent and agreed to participate in the study.

### 2.1. Animals

Subjects were selected from an initial group of 48 dogs whose owners had expressed interest in participating in the study. To be considered for enrollment, dogs had to be either 1–4 years old or over 11 years of age, of either sex and any sexual status and breed, with no known history of clinically relevant medical or behavioral disorders, and not receiving any medications that could influence immune or cognitive function. A physical examination was performed to assess general health, and a blood sample was collected for standard laboratory analyses (i.e., complete blood cell count, serum protein electrophoresis, and clinical chemistry). Additionally, the concentration of C reactive protein (CRP) and of the activity of the antioxidant enzyme paraoxonase 1 (PON-1), which act, respectively, as positive and negative acute phase proteins, were measured using methods validated in dogs [43,44], to exclude the presence of subclinical inflammation. Samples were collected for diagnostic purposes in accordance with standard veterinary procedures and with the owners’ written consent; therefore, no additional ethical approval was required (Regulations of the University of Milan, decision EC 29 October 2012, renewed under protocol No. 02–2016). The results were reviewed by two European College of Veterinary Clinical Pathology (ECVCP) board-certified diplomates. To be eligible for the study, dogs had to be free from clinically relevant signs or existing diagnoses of health disease, including sensory dysfunction and pain, based on both medical history and the clinical examination, and their laboratory results had to be unremarkable. Therefore, four dogs were excluded because, despite the absence of clinical abnormalities, they had moderate anemia, which was associated with neutrophilic leukocytosis in one case. Consequently, the final study sample consisted of 44 dogs: 22 in the 1–4-year-old age group and 22 in the ≥11-year-old group (Table 1), representing young to mature adults and late senior to geriatric individuals, respectively, in line with the age classifications proposed in previous studies [45,46,47]. The two groups were similar in terms of sex, breed type, size, and age at acquisition but differed markedly in sexual status, with desexing being more prevalent among dogs aged 11 years and older. According to the updated size categorization [48], the senior group included 4 dogs classified as I (Toy, 18.2%), 3 as II (Small, 13.6%), 2 as III (Medium, 9.1%), 10 as IV (Large, 45.5%), and 3 as V (Large, 13.6%). No dogs fell into category VI (Giant). The young group included 5 dogs classified as I (Toy, 22.7%), 0 as II (Small, 0.0%), 1 as III (Medium, 4.5%), 8 as IV (Large, 36.4%), 5 as V (Large, 22.7%), and 3 as VI (Giant, 13.6%). As expected based on the selection criteria, the clinicopathological screening, including immunoglobulin G (IgG) and immunoglobulin M (IgM), assessed as indicators of potential immune activation, did not reveal any values outside the reference intervals in either age group, despite a marginally significant (*p* = 0.040) trend toward lower hematocrit (Ht) values and a significantly higher concentration of globulin fractions in older animals, resulting in a reduced albumin-to-globulin ratio, as previously reported in the literature [15,49,50]. Overall, dogs in both groups could thus be considered free from subclinical inflammation, as indicated by the hematological and biochemical indicators of inflammation reported in Supplementary Table S1.

### 2.2. Personality and Cognitive Scales

The following questionnaires were completed by caregivers using a secure online link. Each questionnaire was linked to the corresponding canine subject through a unique code provided with the access link. The responses were scored by a researcher who was blind to the dogs’ identity and age. Final scoring was reviewed and supervised by a resident of the European College of Animal Welfare and Behavioural Medicine (ECAWBM).

#### 2.2.1. Dog Impulsivity Assessment Scale (DIAS)

The DIAS consists of 18 items measuring on a five-point scale, from Strongly Agree (5) to Strongly Disagree (1), an overall questionnaire score (OQS), and three independent factors [51] that reflect distinct aspects of impulsivity and environmental reactivity: Factor 1, “Behavioral Regulation” (a high score implies higher trait impulsivity), Factor 2, “Aggression and Response to Novelty” (a high score suggests a more aggressive/aversive aversion to novelty) and Factor 3, “Responsiveness” (a high score implies fast and engaged responses to new things).

#### 2.2.2. Reinforcement Sensitivity Theory Personality Questionnaire-Dog (RSTPQ-D)

The psycho-biologically derived Dog-RSTPQ [39] used was recently validated in Italian [38]. It consists of 21 items on a five-point Likert scale, assessing three core motivational systems which describe the three RST domains: the Behavioral Activation System (BAS; reward sensitivity and exploration), the Fight/Flight/Freeze System (FFFS; fear and avoidance), and the Behavioral Inhibition System (BIS; anxiety and conflict monitoring). A high score indicates high sensitivity to that domain, while a low score indicates low sensitivity.

#### 2.2.3. Canine Cognitive Assessment Scale (CCAS)

The CCAS scale used to assess the dog’s cognitive health [42] comprised 17 items that assessed six domains of behavior (disorientation, sleep–wake cycles, social interactions, learning and memory, activity level, and anxiety). The scale utilized a four-point Likert scale, reporting behavior over the previous six months.

### 2.3. Cytokine and Immunoglobulin Measurements

For each dog, the serum concentrations of the pro- and anti-inflammatory cytokines (TNF-α, IL-6 and IL-10) and of immunoglobulin-G (IgG) and immunoglobulin-M and (IgM) were measured using ELISA assays. Specifically, after routine laboratory workout, serum leftover samples were immediately stored at −20 °C upon collection and stored for up to six months without being thawed. Upon analysis, the samples were thawed at room temperature and assessed using commercially available enzyme-linked immunosorbent assay (ELISA) kits specifically designed for the quantitative determination of TNF-α, IL-6, IL-10, IgG, and IgM in dogs. Samples were diluted 1:2 or 1:4 based on pre-experiment results and the manufacturer’s recommendations. Information regarding the ELISA kits used in this study and their analytical performances is summarized in Supplementary Table S2. Each sample was prepared in duplicate, and concentrations were determined using a Micro Read 1000 microplate reader (Global Diagnostic, Geel, Belgium) in accordance with the relevant standard curves.

### 2.4. Statistical Analysis

All statistical analyses were conducted using SPSS version 29.0 (IBM SPSS Statistics for Mac, Armonk, NY, USA). For the RSTPQ-D, domain scores were calculated as follows: FFFS = mean of items 1–7, BIS = mean of items 8–14, and BAS = mean of items 15–21. No reverse scoring was required. Dogs were classified as high or low scorers in each domain based on whether their score was above or below the theoretical midpoint (i.e., 3.0 on a 1–5 scale). In line with the original scoring method [51], DIAS scores were computed by summing all valid responses and dividing by five times the number of items answered (since five is the maximum item score), producing an overall questionnaire score (OQS, range 0–1), where higher scores indicate greater impulsivity or reduced self-control. Each DIAS sub-factor was calculated as a ratio of the potential total score for the items completed. As for the CCAS, the total score was calculated by summing all items. Final scores were interpreted according to established thresholds: 0–7 = normal aging, 8–40 = mild to moderate cognitive impairment, and 41–69 = severe impairment [42].

Given that several variables were ordinal scores, non-parametric tests were used to ensure consistency across analyses. Descriptive statistics for categorical variables were derived from 2 × 2 contingency tables and compared across age groups using Pearson’s chi-square test of independence with continuity correction; Fisher’s exact test was applied when expected cell counts were below 5. Continuous variables were compared using the independent-samples Mann–Whitney U test.

Preliminary Spearman’s rank correlations were conducted on the full sample (n = 44) to explore overall cross-domain associations (i.e., inflammation–personality and inflammation–cognition) and to identify redundancies within each domain, prior to multivariate analysis aimed at examining relevant immune–behavioral profiles. The strength of correlation was classified as absent (0.00–0.09), weak (0.10–0.29), moderate (0.30–0.49), or strong (0.50–1.00) [52]. Associations with *p* ≤ 0.05 were retained; however, when two variables within a domain were highly correlated (ρ ≥ 0.80), one was excluded to avoid multicollinearity. Personality traits or cognitive scores showing significant associations in these preliminary analyses were identified as candidate outcome variables for subsequent regression models. These candidate outcomes were then tested for between-group differences (i.e., age group, sex, sexual status, breed type, and source) using the Mann–Whitney U test. These preliminary analyses were conducted as part of a conservative strategy to guide variable selection and reduce the risk of overfitting. Except for age group, which was always included to explore potential age-related patterns and interactions, only variables meeting at least one inclusion criterion (significant cross-domain correlation without multicollinearity or significant group difference) were entered into generalized linear models (GzLMs) as independent variables. No corrections for multiple testing were applied, as this was a bivariate pre-screening of candidate explanatory variables, aimed at informing variable selection in subsequent models.

Gamma models with a log link function were used for continuous or quasi-continuous outcomes, while Poisson loglinear models were applied to ordinal data. Separate models were run for each behavioral domain of interest, to explore the potential modulatory role of inflammatory biomarkers as a function of age, while preserving interpretability. Each model initially included all main effects and interaction terms. The most parsimonious model was then selected based on the lowest Akaike Information Criterion (AIC), with non-contributing predictors removed. Associations were expressed as odds ratios (Exp[B] in SPSS) with 95% confidence intervals (CIs), and *p*-values ≤ 0.05 were considered statistically significant.

## 3. Results

Descriptive statistics for DIAS and RSTPQ-D personality scores and serum cytokine concentrations are reported in Table 2 and Table 3.

**Figure 1:**
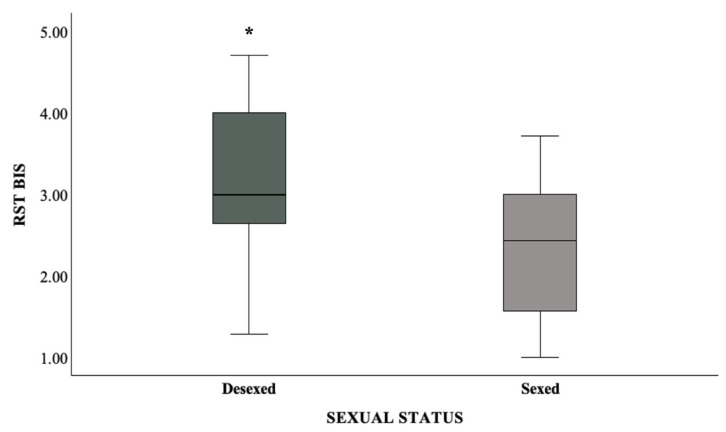
Clustered boxplot displaying BIS scores by sex status (desexed vs. sexed) in the entire sample of dogs (44). * = Independent-samples Mann–Whitney U test, *p* = 0.008.

The CCAS revealed either normal aging or only mild cognitive impairment, with no differences between the two age groups (1–4 year-old age group: median score = 0.00, min = 0, max = 15; ≥11 year-old group: median score = 3.50, min = 0, max = 36; Fisher Exact test *p* = 1.000). Preliminary correlation analyses confirmed the absence of significant multicollinearity within behavioral or physiological domains. Moderate significant correlations emerged between TNF-α and FFFS (ρ = −0.337, *p* = 0.039) and between IL-10 and BIS (ρ = −0.436, *p* = 0.006), supporting the inclusion of these variables in the regression models. A significant positive correlation was also found between BIS and FFFS (ρ = 0.447, *p* = 0.037). In contrast, IL-6 did not show significant correlations with any personality traits or cognitive scores and was therefore excluded from subsequent analyses. No statistically significant differences emerged among age groups for any of these variables. A non-parametric independent-samples Mann–Whitney U test revealed a significant difference in the distribution of BIS scores between desexed and sexed dogs (*p* = 0.008), with desexed individuals exhibiting higher scores (Figure 1). This finding supported the inclusion of sex status as a covariate in the regression models to account for its potential confounding effect.

The results from the GzLM are summarized in Table 4, which reports only statistically significant factors. A generalized linear model was performed with FFFS (fear/avoidance) as the dependent variable and TNF-α, age group, BIS (behavioral inhibition), and the interaction term TNF-α × age group as predictors. The overall model was significant (*p* = 0.001), and model fit was acceptable (AIC = 88.368; deviance/df = 0.106). A very small but significant effect (*p* = 0.001) of the interaction between TNF-α and age group was found, indicating that the relationship between TNF-α and FFFS differed by age. Specifically, in late senior–geriatric dogs, higher TNF-α concentrations significantly predicted lower FFFS scores, with a 0.1% decrease in FFFS for each 1 pg/mL increase in TNF-α (Exp[B] = 0.999, 95% CI: 0.999–1.000). There was also a significant (*p* = 0.002) and positive relationship between BIS and FFFS in late senior–geriatric dogs, with the odds of a higher FFFS score increased by 24% for each one-unit increase in BIS score (Exp[B] = 1.240, 95% CI: 1.080–1.420). No significant main effect of age group or TNF-α alone was observed, and no significant associations were detected in young dogs. A generalized linear model was performed with BIS as the dependent variable and IL-10, age group, IL-10 × age group interaction, sex status, and FFFS as predictors. The overall model was significant (*p* = 0.001), and model fit was acceptable (AIC = 99.90; deviance/df = 0.090). The IL-10 × age group interaction was significant (*p* = 0.001), suggesting a small relationship between IL-10 and BIS as a function of age stage. Specifically, in late senior–geriatric dogs, higher IL-10 levels significantly predicted lower BIS scores, indicating a 2.2% decrease in behavioral inhibition per unit (pg/mL) increase in IL-10 (Exp[B] = 0.978, 95% CI: 0.968–0.989). In contrast, age group and IL-10 did not reach statistical significance as main effects, and no significant associations were detected in young dogs.

A significant main effect of sex status was found (*p* = 0.028), indicating that desexed dogs exhibited higher BIS scores than sexually intact dogs. Specifically, the odds of increased BIS scores were approximately 28% greater in desexed dogs (Exp[B] = 1.283, 95% CI: 1.027–1.603).

## 4. Discussion

In the present study, we found small but significant (*p* = 0.001) predictive effects of serum TNF-α and IL-10 levels on fear- and anxiety-related personality traits, moderated by age. Specifically, in the late senior–geriatric age group, higher TNF-α levels predicted lower Fight/Flight/Freeze System (FFFS) scores, with a 0.1% decrease per pg/mL increase, whereas higher IL-10 levels predicted lower Behavioral Inhibition System (BIS) scores, corresponding to a 2.2% decrease per pg/mL increase. No significant relationships were found in younger dogs. The neural and immune systems are known to be intrinsically intertwined [53]. So, while it is possible that the observed covariations between immune biomarkers and behavioral traits arose by chance or resulted from mechanisms that affect both systems simultaneously (e.g., genetic and/or epigenetic modulators), without reflecting a direct physiological link, it is likely that at least part of this covariation could be explained by some aspect of the underlying physiology [53]. Theoretically assuming a causal relationship, our results align with recent affective immunology research positing that the immune system might control personality traits which are related to adaptive behaviors, rather than vice versa [54].

The RST FFFS involves the activation of the amygdala and periaqueductal grey (PAG) [55,56] which coordinate the appropriate defensive responses to fearful or threatening events [57]. The FFSS is in fact responsible for personality traits such as proneness to fear [58], with high sensitivity resulting in individuals being prone to interpret novel stimuli as dangerous, or anyway punishing, and engaging in defensive avoidance strategies traditionally classified as active (fight/flight) or passive (freezing), based on the perceived intensity of the threat [59,60]. More recently, however, freezing has been reconceptualized as an active preparatory state that functions as a parasympathetic brake on the motor system, enhancing sensory processing and action readiness [61]. According to Roelofs (2017), this state is modulated not only by the amygdala and PAG but also by hormonal dynamics, particularly the cortisol–testosterone balance, which influences both the expression and threshold of freezing behavior [61].

It is important noting that, in the Dog-RST questionnaire, the FFFS component primarily captures the “flight” and “freezing” responses but lacks coverage of aggression-related behaviors (e.g., barking or growling). This is why we included the DIAS questionnaire, which specifically addresses this aspect with the “Aggression and response to novelty factor”, making the assessment more comprehensive [39].

The fearfulness trait exhibits strong psychoneuroimmunologic connections to the highly conserved biological process of inflammation [62], which, together with the display of defensive responses to aversive stimuli [63], contributes to the maintenance or restoration of homeostasis through allostatic mechanisms [12,64]. Thus, at first glance, our finding that higher TNF-α concentrations predict lower sensitivity to fear and avoidance behaviors (i.e., lower FFFS scores) might seem counterintuitive, as heightened inflammation could be expected to be linked to increased sensitivity to threat [65]. However, not all reports in the scientific literature indicate a positive association between inflammation and fear-related responses [66,67], highlighting the complexity of immune–behavior relationships, which are likely influenced by multiple factors, including age and life-course exposures [68,69]. For instance, increases in TNF-α levels may reflect an activated immune system, which could in turn reduce the organism’s reliance on behavioral avoidance strategies, leading to diminished sensitivity to defensive responses. This immune system–brain interaction becomes phenotypically apparent as a “behavioral immune response” [70,71]. The behavioral immune response theory proposes that individuals with more efficient immune defenses may not need to engage in pronounced harm-avoidant behaviors to maintain homeostasis. In our study, the observed inverse association between TNF-α and FFFS in older dogs (i.e., higher inflammation predicting lower fear and avoidance traits) may therefore be interpreted as a potentially adaptive recalibration. Specifically, if increasing TNF-α levels represent a biologically protective state in healthy subjects, such as the clinically healthy dogs in our study, then reduced sensitivity in the defensive system (lower FFFS scores) could reflect a decreased need for behavioral defense under conditions of increased immune surveillance. These dynamics appear consistent with a well-regulated response; however, both inflammation and fearfulness trait can be either well-regulated, thus promoting adaptive function, or become dysregulated over time and contribute to dysfunction [72]. Individuals are exposed to stressors, whether real or perceived, and the associated inflammatory response throughout life [73]. The cumulative effects of repeated real or perceived stressors and relatively low-level bouts of inflammation can lead to dysregulation of both HPA axis and immune function. This promotes the chronic, low-grade inflammation characteristic of aging (inflammaging) and drives the vulnerability of older animals to both physical and emotional stressors [74]. More precisely, it has been proposed that systemic low-grade inflammation may affect personality by depleting cognitive, emotional, and physical resources that would be needed to effectively cope with stressful situations through adaptive behaviors [75]. In the context of chronic low-grade inflammation, as seen in aging, elevated TNF-α may reflect an ongoing dysregulation, rather than efficient immune function. This suggests that our findings might also represent a maladaptive blunting of defensive responses in the context of inflammaging, resulting in a dysregulated avoidance response that is diminished even when potential risks are present, possibly increasing the likelihood that vulnerable individuals struggle to cope with low-level or routine stressors. It is worth noting that late senior–geriatric dogs displayed no clinically relevant signs and did not differ significantly from younger dogs in TNF-α/IL-6 levels, despite higher medians suggesting a general trend toward increased concentrations with age. The cytokine concentrations followed age-related trends previously reported in healthy dogs consistent with their clinically healthy status for their age [14]. This suggests that the observed immune–personality association is more likely to represent a subtle, rather than overt, and possibly early signal of dysregulation emerging in the context of low-grade chronic inflammation (inflammaging). The observed 0.1% decrease in fear/avoidance scores per unit increase in TNF-α may appear minimal; however, the broader interquartile range (IQR) observed in late senior–geriatric dogs suggests substantial individual variability in pro-inflammatory cytokine levels. This heterogeneity may attenuate statistical associations in relatively small samples, potentially explaining both the limited effect size of TNF-α and the absence of significant associations for IL-6, which followed a similar trend but displayed even greater variability. Still, within the framework of inflammaging, characterized by a silent and uneven rise in pro-inflammatory markers with advancing age, even small neuroimmune shifts may alter the threshold for defensive responding. Accordingly, this pattern, suggestive of a reduced capacity for functional defensive responses in the face of threat, may reflect a biologically meaningful, latent dysregulation in immune–behavioral functioning during aging, signaling vulnerability before overt dysfunction emerges. A better understanding of these subtle dynamics could ultimately support the development of earlier and more sensitive tools for identifying age-related risks in clinically healthy dogs.

Fearfulness is closely linked to another survival-evolved mechanism, namely, anxiety, with individuals who are more prone to fear responses also engaging more readily in risk assessment and behavioral hesitation [76]. As conceptualized in the RST, the BIS is rooted in the limbic system and reflects a vigilance system responsible for maintaining caution, low impulsivity [39], and heightened monitoring for potential threats in ambiguous situations [77]. It is therefore not surprising that, among the dogs in the late senior–geriatric group, BIS sensitivity positively predicted FFFS sensitivity, with the odds of a higher FFFS score increasing by 24% for each one-unit increase in BIS score. In apparent contrast, higher serum concentrations of IL-10 were predictive of lower BIS sensitivity in this age group. However, according to the behavioral immune system theory [71], this finding might still reflect a protective strategy. When individuals perceive themselves as highly vulnerable, as may be the case in aging dogs, they tend to become more sensitive to potential threats [71]. This heightened vigilance predisposes them to chronic anxiety and stress, which may accelerate aging through inflammaging and increase the risk of chronic inflammatory comorbidities [78]. Supporting this notion, increased anxiety is one common report in aged humans and dogs, and is a recognized symptom of both Alzheimer’s disease [79] and Canine Cognitive Dysfunction (CCD) [21]. Anxiety can also be triggered by pain [80], to which older dogs are more susceptible due to age-related conditions such as osteoarthritis [37]. High sensitivity to BIS predicts individual predisposition for anxiety [38] and, in our study, dogs aged 11 years and older exhibited slightly—but not significantly—higher BIS scores compared to younger dogs. The behavioral immune system model assumes that the activation of the vigilance system is inversely proportional to an individual’s subjective perception of vulnerability to infection, emotional stress, or other biological challenges. In this model, personality traits such as anxiety are functionally and flexibly modulated in response to this perceived vulnerability, linking immune function and behavior in ways that support individual protection. IL-10 is an anti-inflammatory cytokine primarily known for its anti-inflammatory and neuroprotective properties, exerted through different signaling pathways [81,82]. It may therefore affect brain function and behavior through immune regulation, impacting various affective states, including anxiety [83]. Experimental studies in rodents support this modulatory role: IL-10 attenuates the inflammation-induced behavioral changes, including anxiety-like responses [84], while IL-10 knockouts display hyperanxious behaviors [85]. Within this framework, the observed reduction in anxiety-related traits with higher serum concentrations of IL-10 in older dogs may not necessarily reflect maladaptive emotional disengagement or dysfunction. Although this possibility cannot be completely ruled out, especially considering the pleiotropic nature of IL-10 [86], which may exert context-dependent effects, including potentially opposing actions [83], it may instead signal a protective downregulation of anxiety aimed at minimizing unnecessary activation in healthy aging individuals. Overall, depending on the adaptive or maladaptive function, this association between higher IL-10 concentrations and lower BIS scores could point to IL-10 as a candidate marker of emotional resilience in senior dogs or be of practical value for early identification of individuals at greater risk for anxiety-related dysregulation in aging. In any case, if confirmed, this link may carry functional relevance for the development of targeted interventions to support emotional health in older dogs.

Interestingly, in the study by Mesquita et al. (2008) [85], the authors excluded any influence of IL-10 on cognitive performance, a finding that mirrors our observation, where IL-10 predicted variation in RST behavioral inhibition but was unrelated to cognitive outcomes (CCAS scores). Similarly, in our study, TNF-α and IL-6 also showed no correlation with cognitive measures. This may be at least partially explained by the fact that dogs in our sample exhibited either normal aging or only mild cognitive impairment, regardless of age, and therefore may not have reached the threshold of dysfunction at which immune–cognition interactions become detectable.

Lastly, it seems worth mentioning that, between the two age groups, desexed dogs were significantly more likely to exhibit higher BIS scores compared to their intact counterparts. This finding agrees with accumulating evidence that desexing, while commonly performed to mitigate certain problem behaviors, including some forms of aggression, may also be associated with increased fearfulness, anxiety, and stress-related behaviors—emotional states that can, in turn, contribute to the expression of aggressive behaviors [87,88]. Of course, it is important to clarify that neutering in companion dogs is also commonly carried out for reasons beyond behavioral management, including population control and the prevention of issues associated with reproductive behavior (e.g., estrus, roaming, and mating attempts). The procedure is often promoted as part of responsible pet ownership and is routinely performed in shelters to help address overpopulation [87]. From a neurobiological perspective, the removal of gonadal hormones such as testosterone and estrogen can disrupt the regulatory balance among several key neuromodulators, particularly cortisol, oxytocin, serotonin, and dopamine, which influence emotional stability and behavioral flexibility [87]. For example, lower oxytocin binding following gonadectomy may reduce personality traits related to social confidence and increase dimensions associated with threat sensitivity [88]. Moreover, the potential increase in cortisol-to-testosterone ratios post-neutering may further amplify stress reactivity and anxiety-related traits [88,89]. As already cited, the BIS is activated in contexts of uncertainty and goal conflict, and is behaviorally expressed through caution, vigilance, and heightened anxiety. The higher BIS score observed in desexed individuals in our study may therefore reflect a greater predisposition toward risk-avoidant or anxious responses. Interestingly, Sundburg et al. (2016) [90] reported that neutered female dogs exhibit an increased risk of developing an autoimmune condition, namely lupus erythematosus (LUP), supporting the hypothesis that gonadectomy may affect not only neuroendocrine regulation but also immune system function. These findings underscore the importance of investigating the complex, multidimensional interactions involving immune, hormonal and behavioral systems, an integrative perspective that forms the basis of the present study.

## 5. Conclusions

To sum up, these findings suggest that emotional aging in dogs may reflect a nuanced interplay between distinct reactive and immunoregulatory processes and individual behavioral traits. The cross-sectional design, the reliance on correlational and regression analyses, and the relatively small sample size do not allow the demonstration of causal inferences on the direction of the observed negative relationships among TNF-α, IL-10, and fear- (FFFS) and anxiety-related (BIS) traits in late senior and geriatric dogs. To determine whether these immune–behavior associations reflect true causal pathways or are merely correlative, future research should employ prospective, blinded longitudinal designs with repeated measures and mechanistic approaches.

Nevertheless, these preliminary results may point to novel pathways through which the immune system interacts with personality in aging animals, potentially signaling—or even influencing—resilience or vulnerability to emotional dysregulation. The two immune–personality associations (TNF-α with FFFS and IL-10 with BIS) found in the late senior–geriatric group may, in fact, have either adaptive or maladaptive implications (see Table 5 for a summary of possible interpretations). These associations could reflect resilience-promoting mechanisms, such as the protective downregulation of fear and anxiety traits in healthy aging individuals. Alternatively, they might represent maladaptive responses or ineffective compensatory processes, potentially signaling an altered capacity for functional defensive and vigilance responses. This emotional disengagement could, in turn, contribute to increased susceptibility to inflammaging and emotional dysregulation. It is also possible that these patterns diverge, one compensating for the vulnerability conferred by the other, or even shift over time within individuals, depending on environmental and internal factors, such as hormonal changes, stress exposure, comorbidities, or genetic predispositions [53]. The potential variability emphasizes the importance of interpreting these associations not as fixed outcomes, but as part of flexible dynamics. Clarifying these dynamics through longitudinal and mechanistic studies should be a priority for future research, as they may be critical for distinguishing resilient from at-risk aging trajectories and for informing early, individualized interventions to support emotional well-being in aging dogs. Future investigations should consider incorporating additional markers of inflammation beyond the cytokines measured in the present study, as well as integrating behavioral and physiological assessments of chronic stress—such as hair cortisol measurement—thus overcoming two key limitations of the current work. More broadly, our results offer insights into how immune and personality factors may interact in later life stages, with possible translational relevance to aging-related emotional challenges in humans and other species. In this context, the domestic dog emerges as a particularly valuable model in gerontology, given the greater similarity of their life history and aging processes to those of humans and other great apes compared to laboratory rodents.

## Figures and Tables

**Table 1:** Main demographic characteristics of each age-subgroup.

Age Group (N)	Median Age (min–max) (Years)	Sex	Desexed (%)	Purebred (%)	Age at Acquisition	Main Sources
		F/M			0–2 months/ 3–5 months/ 6–12 months/>12 months	Homebred/friends-relative/pet shop-online/registered breeder/shelter-rescue/stray
1–4 years old (22)	3 (1–4)	11/11	41	41	11/5/3/3	6/6/0/4/5/1
≥11 years old (22)	11 (11–18)	9/13	86 *	50	7/6/1/8	0/6/3/7/5/1

F = female; M = male. * = Pearson’s chi-square test with continuity correction, *p* = 0.005.

**Table 2:** The descriptive statistics of the personality variables in this study.

Age Group		DIAS Factor 1	DIAS Factor 2	DIAS Factor 3	DIAS Total Score	RST FFFS	RST BIS	RST BAS
1–4 years old	Median	0.42	0.36	0.76	0.61	2.57	2.64	4.07
	Minimum	0.24	0.20	0.48	0.47	1.00	1.00	1.57
	Maximum	0.64	0.60	1.00	0.77	4.43	4.57	5.00
≥11 years old	Median	0.40	0.52	0.64	0.54	2.00	3.00	3.21
	Minimum	0.22	0.20	0.36	0.42	1.00	1.00	1.00
	Maximum	0.64	0.76	0.88	0.66	3.57	4.71	5.00

DIAS Factor 1: Behavioral Regulation; DIAS Factor 2: Aggression and Response to Novelty, DIAS Factor 3: Responsiveness. RST FFFS: Fight/Flight/Freeze System; RST BIS: Behavioral Inhibition System; RST BAS: Behavioral Activation System.

**Table 3:** The descriptive statistics of the serum cytokine concentrations.

	Group	
Cytokine	Young	Senior
	Median	
	IQR (25th–75th percentile)	
TNF (pg/mL)	14.40 52.07 (2.10–54.17)	43.303 103.31 (9.06–112.37)
IL10 (pg/mL)	24.27 26.12 (8.38–34.50)	16.28 24.08 (6.37–30.45)
IL6 (pg/mL)	0.74 167.21 (0.10–167.31)	9.44 3027.22 (0.10–3037.32)

IQR = interquartile range.

**Table 4:** Generalized linear model predicting the RST BIS score.

Parameter	B	Std. Error	Sig.	Exp(B)	95% Wald Confidence Interval for Exp(B)	
					Upper	Lower
**Dependent Variable: BIS**						
[Age group = LS-G] * IL-10	−0.022	0.005	0.001	0.978	0.968	0.989
[Sex status = Desexed]	0.249	0.114	0.028	1.283	1.027	1.603
**Dependent Variable: FFSS**						
[Age group = LS-G] * TNF-α	−0.001	0.002	0.001	0.999	0.999	1.000
[Age group = LS-G] * BIS	0.215	0.068	0.002	1.240	0.980	1.332

Age group: LS-G: late senior–geriatric (≥11 years old).

**Table 5:** Possible interpretations of immune–personality associations observed in late senior–geriatric dogs.

Cytokine	Reinforcement Sensitivity Theory (RST) Trait	Observed Association	Adaptive Interpretation	Maladaptive Interpretation	Note
TNF-α	FFFS	↓ FFFS ↑ TNF-α	Protective downregulation of threat responses to reduce metabolic cost and emotional burden in older, vulnerable individuals, possibly reflecting a compensatory recalibration during inflammaging	Neuroinflammation or chronic immune activation impairs functional defensive circuits, leading to emotional disengagement or reduced vigilance	The co-variation may be either casual or reflect concomitant components of broader adaptive or maladaptive profiles associated with aging
IL-10	BIS	↓ BIS ↑ IL-10	Modulation of emotional responses by suppressing excessive anxiety, promoting resilience to stress and enhancing regulatory balance	Overregulation may dampen necessary caution, possibly reflecting early signs of immune–behavioral dysregulation	

FFSS = Fight/Flight/Freeze System; BIS = Behavioral Inhibition System; ↓ = decrease; ↑ = increase.

## Data Availability

Data will be made available upon reasonable request.

## Supplementary Materials

The following supporting information can be downloaded at https://www.mdpi.com/article/10.3390/ani15162418/s1: Table S1: Hematological and biochemical indicators of inflammation in the two age groups of dogs in the study; Table S2: ELISA kits used in the study and their analytical performance characteristics.
