# Supplementary material for: Serum TNF-Alpha and IL-10 Predict Reduced Sensitivity to Fear- and Anxiety-Related Traits in Healthy Older Dogs: Preliminary Evidence for Immune–Personality Signatures in Later Life
Source: Animals (Basel). 2025 Aug 18;15(16):2418. doi: 10.3390/ani15162418 (PMC12383112; doi:10.3390/ani15162418)
Supplement: Supplementary file 1 [file animals-15-02418-s001.zip › animals-3781147-supplementary.pdf]

Supplementary Table S1

Hematological and biochemical indicators of inflammation in the two age groups of dogs in the study.

	GROUP		Independent-Samples Mann–Whitney U Test	Reference Interval
**Parameter**	**Young**	**Senior**		
	Median<br>IQR (25th–75th percentile)		P	
Ht (%)	49.45<br>7.38 (44.80-52.17)	45.3<br>7.9 (41.05-48.95)	0.040*	37.00-57.00
WBC (×10³/μL)	8.69<br>10.07 (7.69-11.30)	8.53<br>4.86 (7.37-12.23)	0.907	6.00-13.90
Neut (×10³/μL)	5.23<br>2.91 (4.17-7.07)	5.48<br>2.71 (4.54-7.26)	0.606	3.00-11.50
Lym ×10³/μL	2.30<br>0.93 (2.04-2.97)	2.03<br>1.04 (1.68-2.72)	0.166	1.00-4.80
Mono (×10³/μL)	0.59<br>0.32 (0.45-0.77)	0.62<br>0.42 (0.41-0.83)	0.994	0.10-1.50
CRP (mg/L)	0.00<br>3.01 (0.00-3.01)	0.00<br>3.47 (0.00-3.47)	0.962	< 5.00
PON-1 (U/L)	238.00<br>75.50 (205.75-281.25)	245.00<br>53.00 (222.00-275.00)	0.805	> 116.00
IgG (ng/mL)	147.56<br>62.51 (120.00-182.51)	159.16<br>145.55 (131.87-277.42)	0.317	ND
IgM (ng/mL)	1.00<br>1.73 (0.68-2.41)	1.30<br>2.28 (0.53-2.81)	0.828	ND
Total Protein (g/dL)	6.20<br>0.35 (5.98-6.33)	6.60<br>1.05 (6.20-7.25)	0.005*	5.40-7.50
Alb (g/dL)	3.34<br>0.43 (3.14-3.57)	3.18<br>0.72 (2.95-3.67)	0.577	2.40-4.70
Alpha1 (g/dL)	0.17<br>0.02 (0.16-0.18)	0.20<br>0.06 (0.17-0.22)	0.056	0.10-0.30
Alpha2 (g/dL)	0.92<br>0.20 (0.83-1.03)	1.15<br>0.35 (1.01-1.35)	0.001*	0.60-1.30
Beta1 (g/dL)	0.22, 0.06<br>(0.21-0.27)	0.27<br>0.06 (0.25-0.31)	0.006*	0.20-0.70
Beta2 (g/dL)	0.88<br>0.28 (0.77-1.04)	1.23<br>0.32 (0.99-1.32)	0.001*	0.50-1.30

Gamma (g/dL)	0.53<br>0.29 (0.42-0.71)	0.64<br>0.23 (0.53-0.76)	0.085	0.40-0.90
A/G	1.17<br>0.33 (1.05-1.39)	0.96<br>0.17 (0.86-1.03)	0.001*	0.80-1.65

Notes: IQR = interquartile range; Ht = hematocrit; WBC = total white blood cell count; Neut = neutrophils; Lym = lymphocytes; Mono = monocytes; CRP = C-reactive protein; PON-1 = paraoxonase-1; IgG 0 immunoglobulin G; IgM = immunoglobulin G; Total Protein = total protein concentration; Alb = albumin; Alpha1 = α1-globulin fraction; Alpha2 = α2-globulin fraction; Beta1 = β1-globulin fraction; Beta2 = β2-globulin fraction; Gamma = γ-globulin fraction; A/G = albumin-to-globulin ratio; * statistical significance = p ≤ 0.05.

Supplementary Table S2

ELISA kits used in the study and their analytical performance characteristics.

Target molecule	Manufacturer	Code	Intra-assay imprecision (CV)	Inter-assay imprecision (CV)	Detection range	Analytical sensitivity
TNF-α	AssayGenie, Dublin, Ireland	CNFI00020	< 8%	< 10%	15.6-1000.0 pg/mL	9.37 pg/mL
IL-6	Biorbyt, Cambridge, UK	orb561401	< 5.98%	< 5.15%	31.2-2000.0 pg/mL	18.75 pg/ml
IL-10	AssayGenie, Dublin, Ireland	CNDL00042	< 10%	< 12%	15.6-1000.0 pg/mL	5.5 pg/mL
IgG	Biorbyt, Cambridge, UK	orb561399	< 5.12%	< 5.41%	0.78-50.00 ng/mL	0.469 ng/mL
IgM	Biorbyt, Cambridge, UK	Orb2667033	< 8%	< 10%	7.81-500.00 ng/mL	4.69 ng/mL
